# Programmed cell death 1 expression is associated with inferior survival in patients with primary central nervous system lymphoma

**DOI:** 10.18632/oncotarget.20264

**Published:** 2017-08-14

**Authors:** Hyunsoo Cho, Se Hoon Kim, Soo-Jeong Kim, Jong Hee Chang, Woo-Ick Yang, Chang-Ok Suh, Yu Ri Kim, Ji Eun Jang, June-Won Cheong, Yoo Hong Min, Jin Seok Kim

**Affiliations:** ^1^ Division of Hematology, Department of Internal Medicine, Yonsei University College of Medicine, Severance Hospital, Seoul 03722, Republic of Korea; ^2^ Graduate School of Medical Science and Engineering, Korea Advanced Institute of Science and Technology, Daejeon 34141, Republic of Korea; ^3^ Department of Pathology, Yonsei University College of Medicine, Severance Hospital, Seoul 03722, Republic of Korea; ^4^ Department of Neurosurgery, Yonsei University College of Medicine, Severance Hospital, Seoul 03722, Republic of Korea; ^5^ Department of Radiation Oncology, Yonsei University College of Medicine, Severance Hospital, Seoul 03722, Republic of Korea

**Keywords:** primary central nervous system lymphoma, programmed cell death 1, programmed cell death-ligand 1, programmed cell death-ligand 2, prognosis

## Abstract

Programmed cell death 1 (PD-1) and its ligands PD-L1/PD-L2 have been shown to mediate immune evasion in various cancers, but their prognostic implications in patients with primary central nervous system lymphoma (PCNSL) are poorly understood. Therefore, we analyzed 76 PCNSL patients at initial diagnosis who were treated homogenously with high-dose methotrexate-based chemotherapy, and evaluated the prognostic roles of high immunohistochemical PD-1, PD-L1, and PD-L2 expression. The cut-off values for high PD-1 (≥ 70 cells/high power field [HPF]), PD-L1 (≥ 100 cells/HPF), and PD-L2 (≥ 100 cells/HPF) were determined by the area under the receiver operating characteristic curve. Expression of PD-1, PD-L1, and PD-L2 was high in 7.9%, 13.2%, and 42.1% patients, respectively. High PD-1, (*P* = 0.007) and Memorial Sloan Kettering Cancer Center (MSKCC) prognostic scoring (*P* = 0.019) were independently associated with inferior overall survival on multivariate analysis. High PD-1 also remained an independent prognostic factor for inferior progression-free survival (*P* = 0.028), as did MSKCC prognostic scoring (*P* = 0.041) on multivariate analysis. However, there were no differences in survival according to the expression levels of PD-L1/PD-L2 in PCNSL tumor microenvironment. Our results suggest that PD-1 may be considered a biomarker and potential therapeutic target in PCNSL.

## INTRODUCTION

Primary central nervous system (CNS) lymphoma (PCNSL) is an extranodal non-Hodgkin lymphoma (NHL) confined to the CNS, mostly diffuse large B-cell lymphoma (DLBCL) [1]. Currently, the International Extranodal Lymphoma Study Group (IELSG) and Memorial Sloan Kettering Cancer Center (MSKCC) prognostic scoring systems are the best available clinical tools to risk-stratify patients with PCNSL [2, 3]. However, these prognostic scores do not take into account underlying tumor biological factors, such as tumor microenvironment of PCNSL. Several biomarkers for PCNSL have been suggested, including multiple myeloma oncogene 1/interferon regulatory factor 4 (MUM1/IRF4), B-cell lymphoma-6 (BCL-6) and CD68, but their major limitation is that it is difficult to use those biomarkers as a potential therapeutic target [4–6]. Therefore, discovering a novel biomarker that supplements IELSG and MSKCC prognostic scoring system and that can be used as a therapeutic target is desperately needed to improve prognostication and survival of patients with PCNSL.

Tumor microenvironment per se is important for PCNSL development and progression as well as other malignancies [7]. This is also supported by the study that showed characteristic infiltration by tumor infiltrating lymphocytes in perivascular microenvironment of PCNSL, which was associated with survival [8]. Albeit the immunological role of T-cell infiltration in PCNSL is unclear, emerging data are suggesting that tumors have developed evasion mechanisms that exploit immune checkpoints to overcome antitumor immunity [9].

The immune checkpoint molecule programmed cell death 1 (PD-1) and its ligands PD-L1 and PD-L2 have been shown to play key roles in inhibiting T cell activity in the tumor microenvironment, not only in solid cancers but also in hematologic malignancies [10]. PD-1 and its ligands have been highlighted because their blockade showed outstanding clinical responses in advanced hematologic malignancies [11].

Importantly, PCNSL exhibited frequent 9p24.1/PD-L1/PD-L2 copy number alterations [9] and even PD-1 blockade demonstrated clinical activity in relapsed/refractory PCNSL [12], although only small number of patients were evaluated. Based on these findings, we sought to evaluate PD-1, PD-L1, and PD-L2 expressions in immunocompetent PCNSL patients at diagnosis, as well as their prognostic implication.

## RESULTS

### Patients’ characteristics

Baseline patient characteristics according to PD-1, PD-L1, and PD-L2 expression levels are summarized in Table 1. The median follow-up duration for all patients was 20.2 (range, 2.2–128.5) months, and the median follow-up duration for surviving patients was 31.9 (range, 2.4–128.5) months. Serum Epstein-Barr virus (EBV) DNA was detected in 16 (21.1%) patients (median 9,200 [range 1,944-88,000] copies/mL blood).

**Table 1:** Baseline characteristics of all patients and subgroups according to the levels of PD-1, PD-L1, and PD-L2 expression

Clinical features, *n* (%)	Entire cohort	Subgroups								
	(*n* = 76)	PD-1		*P*	PD-L1		*P*	PD-L2		*P*
		Low (*n* = 70)	High (*n* = 6)		Low (*n* = 66)	High (*n* = 10)		Low (*n* = 44)	High (*n* = 32)	
Median age, year	57 (33-79)	56 (33-79)	59 (34-68)		56 (33-79)	59 (34-64)		58 (36-79)	56 (33-74)	
Age >60	30 (39.5)	28 (40.0)	2 (33.3)	0.556	28 (42.4)	2 (20.0)	0.158	27 (61.4)	19 (59.4)	0.524
Male gender	39 (51.3)	36 (51.4)	3 (50.0)	0.637	35 (53.0)	4 (40.0)	0.334	23 (52.3)	16 (50.0)	0.514
ECOG PS ≥2	35 (46.1)	34 (48.6)	1 (16.7)	0.209	29 (43.9)	6 (60.0)	0.271	17 (38.6)	18 (56.3)	0.099
Elevated serum LDH	34 (44.7)	33 (47.1)	1 (16.7)	0.216	29 (43.9)	5 (50.0)	0.489	20 (45.5)	14 (43.8)	0.535
Deep lesion	51 (67.1)	47 (67.1)	4 (66.7)	0.649	43 (65.2)	8 (80.0)	0.293	27 (61.4)	24 (75.0)	0.158
Elevated CSF protein	30/66 (39.4)	28/61 (40.0)	2/5 (33.3)	0.587	27/57 (40.9)	3/9 (30.0)	0.339	18/39 (40.9)	12/27 (37.5)	0.546
Positive serum EBV	16 (21.1)	14 (20.0)	2 (33.3)	0.371	14 (21.2)	2 (20.0)	0.648	11 (25.0)	5 (15.6)	0.242
Histology, DLBCL/PTCL	75(98.7)/1(1.3)	69(98.6)/1(1.4)	6(100.0)/0(0.0)	0.921	65(98.5)/1(1.5)	10(100.0)/0(0.0)	0.868	43(97.7)/1(2.3)	32(100.0)/0(0.0)	0.579
IELSG				0.257			0.507			0.097
Low (0-1)	12 (15.8)	10 (14.3)	2 (33.3)		10 (15.2)	2 (20.0)		8 (18.2)	4 (12.5)	
Intermediate (2-3)	46 (60.5)	43 (61.4)	3 (50.0)		41 (62.1)	5 (50.0)		29 (65.9)	17 (53.1)	
High (4-5)	8 (10.5)	8 (11.4)	0 (0.0)		6 (9.1)	2 (20.0)		2 (4.5)	6 (18.8)	
Missing	10 (13.2)	9 (12.9)	1 (16.7)		9 (13.6)	1 (10.0)		5 (11.4)	5 (15.6)	
MSKCC				0.180			0.433			0.208
Low (0)	15 (19.7)	13 (18.6)	2 (33.3)		13 (19.7)	2 (20.0)		9 (20.5)	6 (18.8)	
Intermediate (1)	32 (42.1)	29 (41.4)	3 (50.0)		27 (40.9)	5 (50.0)		21 (47.7)	11 (34.4)	
High (2)	29 (38.2)	28 (40.0)	1 (16.7)		26 (39.4)	3 (30.0)		14 (31.8)	15 (46.9)	
Initial treatment MVD/MVP	41(53.9)/35(46.1)	37(52.9)/33(47.1)	4(66.7)/2(33.3)	0.416	40(60.6)/26(39.4)	1(10.0)/9(90.0)	0.003	25(56.8)/19(43.2)	16(50.0)/16(50.0)	0.361
Non-CR1	37 (48.7)	34 (48.6)	3 (50.0)	0.389	31 (47.0)	6 (60.0)	0.562	22 (50.0)	15 (46.9)	0.380
Upfront ASCT	16 (21.1)	15 (21.4)	1 (16.7)	0.629	13 (19.7)	3 (30.0)	0.352	9 (20.5)	7 (21.9)	0.550
Salvage ASCT	10 (13.2)	8 (11.4)	2 (33.3)	0.176	9 (13.6)	1 (10.0)	0.609	7 (15.9)	3 (9.4)	0.318
Consolidation WBRT	12 (15.8)	8 (11.4)	4 (66.7)	0.657	5 (7.6)	7 (70.0)	0.495	7 (15.9)	5 (15.6)	0.384
Salvage WBRT	14 (18.4)	13 (18.6)	1 (16.7)	0.696	12 (18.2)	2 (20.0)	0.590	9 (20.5)	5 (15.6)	0.411

Abbreviations: ECOG, Eastern Cooperative Oncology Group; PS, performance status; LDH, lactate dehydrogenase; CSF, cerebrospinal fluid; EBV, Epstein-Barr virus; DLBCL, diffuse large B-cell lymphoma; PTCL, peripheral T-cell lymphoma; IELSG, International Extranodal Lymphoma Study Group; MSKCC, Memorial Sloan Kettering Cancer Center; MVD, methotrexate, vincristine and dexamethasone; MVP, methotrexate, vincristine, procarbazine and dexamethasone; CR1, complete response after first two cycles of chemotherapy; ASCT, autologous stem-cell transplantation; WBRT, whole-brain radiotherapy.

All the patients received high-dose-methotrexate (HD-MTX)-based chemotherapy as an initial treatment and there were no significant differences in survival according to the initial HD-MTX-based chemotherapy regimens patients received. Sixteen (21.1%) patients received consolidative upfront autologous stem-cell transplantation (ASCT) after a median of 4 cycles (range 2–4) of HD-MTX-based chemotherapy. Ten (16.7%) patients received salvage ASCT. Busulfan plus thiotepa conditioning regimen was used for upfront or salvage ASCT. Twelve (15.8%) patients received consolidation whole-brain radiotherapy (WBRT, median 40.5 [range 25.2–45.0] Gy), and 14 (18.4%) patients underwent salvage WBRT (median 41.4 [range 36.0–54.0] Gy).

Patients did not reach median Overall survival (OS), and the median progression-free survival (PFS) was 18.4 months (95% CI, 9.9–26.8). The two-year OS and PFS rates were 76.3% and 45.4%, respectively.

### Immunohistochemical PD-1, PD-L1 and PD-L2 expression

**Figure 1:**
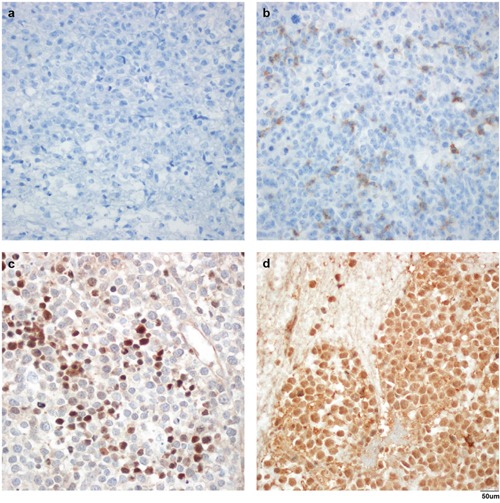
Representative immunohistochemical stains in formalin-fixed and paraffin-embedded samples Negative control **(a)**, high PD-1 expression **(b)**, PD-L1 **(c)**, and PD-L2 **(d)** with anti-PD-1 antibody (NAT105, a and b), anti-PD-L1 antibody (ab58810, c), and anti-PD-L2 antibody (MIH18, d). Magnification, ×400.

Overall, the median PD-1 positive cells/high power field (HPF) was 0 (range, 0–240). Among 76 patients, 41 (53.9%) patients did not show PD-1 positive cells/HPF. However, remaining 35 (46.1%) patients expressed at least one PD-1 positive cells/HPF. The median positive cells/HPF was 10 (range, 0–300) for PD-L1 and 70 (range, 0–300) for PD-L2. Twenty-two (28.9%) and 12 (15.8%) patients did not express PD-L1 and PD-L2, respectively. Remaining 54 (71.1%) and 64 (84.2%) patients expressed at least one PD-L1 and PD-L2 positive cells/HPF, respectively. We stratified patients into high or low PD-1, PD-L1, and PD-L2 expression groups according to the area under curve (AUC)-based cut-off values. Six (7.9%) patients were stratified into the high PD-1 expression group. Ten (13.2%) and 32 (42.1%) patients were stratified into the high PD-L1 and high PD-L2 expression groups, respectively. Representative immunohistochemical stains of PD-1 (Figure 1b), PD-L1 (Figure 1c), and PD-L2 (Figure 1d) are shown in Figure 1 with unstained control (Figure 1a).

### Prognostic factors for survival

On univariate analysis, age > 60 years and Eastern Cooperative Oncology Group (ECOG) performance status (PS) ≥ 2, MSKCC prognostic scoring, IELSG prognostic scoring, and high PD-1 expression level were associated with inferior OS. MSKCC prognostic scoring, patients who did not achieve complete response at initial interim response (non-CR1), patient who did not receive upfront ASCT, and high PD-1 level were associated with inferior PFS also in univariate analysis. On multivariate analysis, high PD-1 (hazard ratio (HR): 4.95, 95% confidence interval (CI): 1.54–15.86, *P =* 0.007) and MSKCC prognostic scoring (HR: 2.56, 95% CI: 1.17-5.64, *P =* 0.019) were independently associated with inferior OS. High PD-1 also remained an independent prognostic factor for inferior PFS, (HR 2.73, 95% CI: 1.12-6.69, *P =* 0.028) as did MSKCC prognostic scoring (HR: 1.56, 95% CI: 1.09-2.45, *P =* 0.041) on multivariate analysis. However, PD-L1 and PD-L2 expression levels were not prognostic in our cohort (Table 2).

**Table 2:** Univariate and multivariate analyses for overall survival (OS) and progression-free survival (PFS)

Clinical features	Univariate analysis		Multivariate analysis			
	*P* for OS	*P* for PFS	HR (95% CI) for OS	*P*	HR (95% CI) for PFS	*P*
Age >60 years	0.038	0.159				
Male gender	0.140	0.707				
ECOG PS≥2	0.047	0.353				
Elevated LDH	0.209	0.103				
Deep brain lesion	0.696	0.361				
Elevated CSF protein	0.074	0.064				
Positive serum EBV	0.148	0.173				
Non-CR1	0.791	0.005				
Non-upfront ASCT	0.238	0.040				
MSKCC scoring	0.029	0.046	2.56 (1.17-5.64)	0.019	1.56 (1.09-2.45)	0.041
IELSG scoring	0.036	0.487				
PD-1 ≥70 cells/HPF	0.018	0.043	4.95 (1.54-15.86)	0.007	2.73 (1.12-6.69)	0.028
PD-L1 ≥100 cells/HPF	0.764	0.793				
PD-L2 ≥100 cells/HPF	0.306	0.940				

OS, overall survival; PFS, progression-free survival; HR, hazard ratio; CI, confidence interval; ECOG, Eastern Cooperative Oncology Group; PS, performance status; LDH, lactate dehydrogenase; CSF, cerebrospinal fluid; EBV, Epstein-Barr virus; CR1, complete response after first two cycles of chemotherapy; ASCT, autologous stem-cell transplantation; HPF, high power field; MSKCC, Memorial Sloan Kettering Cancer Center; IELSG, International Extranodal Lymphoma Study Group.

### Survival according to the expression levels of PD-1, PD-L1, and PD-L2

**Figure 2:**
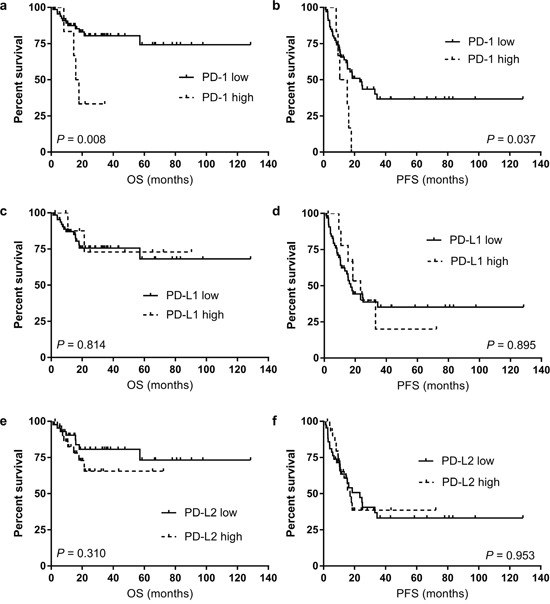
The overall survival (OS) and progression-free survival (PFS) according to the level of PD-1 expression (**a** for OS and **b** for PFS), PD-L1 expression (**c** for OS and **d** for PFS), and PD-L2 expression (**e** for OS and **f** for PFS).

Patients with high expression of PD-1 showed significantly shorter 2-year OS and PFS of 33.3% and 0.0%, in comparison to 81.2% and 50.5% for those with low expression of PD-1 (*P =* 0.008 for OS; Figure 2a, and P *=* 0.037 for PFS; Figure 2b), respectively.

Regarding PD-1 ligands, there were no differences in survival according to the levels of PD-L1 and PD-L2 expression (Figure 2c-2d for PD-L1 and Figure 2e-2f for PD-L2, respectively). Two-year survival rates of patients according to the PD-1, PD-L1, and PD-L2 expression status are provided in Table 3 and associations of PD-1, PD-L1, and PD-L2 expression levels with survival are summarized in Table 4.

**Table 3:** Two-year overall survival of patients according to their PD-1, PD-L1, and PD-L2 expression status

Clinical features, *n*	Entire cohort	Two-year overall survival (%)								
	(*n* = 76)	PD-1		*P*	PD-L1		*P*	PD-L2		*P*
		Low (*n*= 70)	High (*n*= 6)		Low (*n* = 66)	High (*n* = 10)		Low (*n* = 44)	High (*n* = 32)	
Age										
≤60	46	88.7	50.0	0.028	84.5	85.7	0.764	88.6	76.0	0.405
>60	30	66.2	0.0	0.134	61.9	0.0	0.737	66.3	28.6	0.409
Gender										
Male	37	88.0	33.3	0.008	78.2	100.0	0.274	83.3	82.4	0.718
Female	39	74.0	33.3	0.231	73.9	33.3	0.344	81.5	36.0	0.111
ECOG PS										
<2	41	91.6	40.0	0.004	82.0	100.0	0.377	92.4	66.1	0.070
≥2	35	65.9	0.0	0.199	66.2	50.0	0.981	57.9	65.9	0.572
Serum LDH										
Not elevated	42	71.6	40.0	0.162	66.9	66.7	0.754	75.2	50.6	0.243
Elevated	34	89.9	0.0	0.024	87.5	80.0	0.949	87.7	83.6	0.813
Serum EBV DNA load										
Not detected	60	81.7	50.0	0.143	80.4	71.4	0.086	85.4	67.4	0.248
Detected	16	77.4	0.0	0.108	58.0	100.0	0.354	64.6	60.0	0.756
Tumor location										
Non-deep lesion	25	77.6	50.0	0.469	70.6	100.0	0.338	77.4	71.4	0.530
Deep lesion	51	81.4	0.0	0.009	77.9	62.5	0.730	83.1	65.5	0.368
CSF protein										
Not elevated	36	91.8	33.3	0.001	83.5	100.0	0.320	83.3	90.9	0.569
Elevated	30	64.3	50.0	0.673	72.3	33.3	0.440	76.6	30.3	0.249
Not available	10	85.7	0.0	0.155	68.6	100.0	0.558	0.0	60.0	0.295
IELSG										
Low (0-1)	12	77.1	50.0	0.426	65.6	100.0	0.377	75.0	66.7	0.743
Intermediate (2-3)	46	88.9	33.3	0.015	82.4	100.0	0.315	83.2	85.7	0.898
High (4-5)	8	28.1	N/A	N/A	66.7	0.0	0.623	50.0	31.3	0.275
Missing	10	85.7	0.0	0.155	100.0	68.6	0.558	100.0	60.0	0.295
MSKCC										
Low (1)	15	100.0	0.0	<0.001	84.6	100.0	0.571	100.0	66.7	0.070
Intermediate (2)	32	83.9	66.7	0.531	77.6	100.0	0.321	81.5	80.0	0.858
High (3)	29	62.1	0.0	0.333	67.2	0.0	0.235	65.2	33.7	0.468
Initial interim response										
CR1	39	83.6	25.0	0.007	77.6	66.7	0.839	80.8	65.6	0.605
Non-CR1	37	77.0	50.0	0.474	73.8	75.0	0.896	80.9	64.5	0.282
Upfront ASCT										
Yes	16	100.0	0.0	<0.001	92.3	100.0	0.395	100.0	85.7	0.738
No	60	74.4	40.0	0.160	53.3	71.8	0.780	76.2	54.5	0.307
Consolidation WBRT										
Yes	12	66.7	50.0	0.707	70.4	0.0	0.089	79.1	0.0	0.021
No	64	85.4	0.0	<0.001	78.0	100.0	0.159	81.9	80.9	0.962

ECOG, Eastern Cooperative Oncology Group; PS, performance status; LDH, lactate dehydrogenase; EBV, Epstein-Barr virus; CSF, cerebrospinal fluid; IELSG, International Extranodal Lymphoma Study Group; MSKCC, Memorial Sloan Kettering Cancer Center; CR1, complete response after first two cycles of chemotherapy; ASCT, autologous stem-cell transplantation; WBRT, whole-brain radiotherapy; N/A, not available.

**Table 4:** Association of PD-1, PD-L1, and PD-L2 expression with survival

Clinical features	Total patients(*n*)	Deaths(*n*)	Median OS(95% CI)	*P*	Progression(*n*)	Median PFS(95% CI)	*P*
PD-1, cells/HPF				0.008			0.037
<70	70	12	Not reached		34	24.7 (9.8-39.5)	
≥70	6	4	15.8 (11.7-19.9)		6	10.4 (3.3-17.5)	
PD-L1, cells/HPF				0.814			0.895
<100	66	14	Not reached		34	17.9 (6.9-28.8)	
≥100	10	2	Not reached		6	23.4 (13.1-33.8)	
PD-L2, cells/HPF				0.310			0.953
<100	44	8	Not reached		24	23.4 (12.8-33.9)	
≥100	32	8	Not reached		16	17.1 (13.9-20.3)	

OS, overall survival; PFS, progression-free survival; HR, hazard ratio; CI, confidence interval; HPF, high power field.

### Subgroup analysis of patients without upfront ASCT according to PD-1 expression level

**Figure 3:**
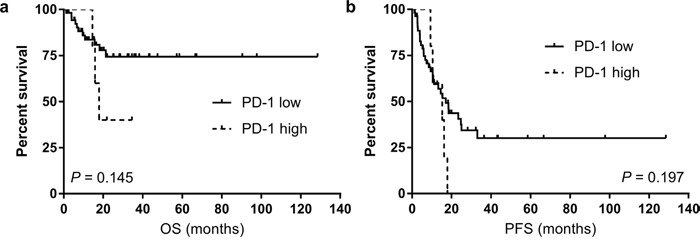
The overall survival (OS, **a**) and progression-free survival (PFS, **b**) according to the level of PD-1 expression among subgroup of patients who did not receive upfront autologous stem-cell transplantation (ASCT).

As high PD-1 expression was independently associated with inferior survival in our whole patients, we performed subgroup analysis in patients who did not undergo upfront ASCT according to the level of PD-1 expression to consider further confounding variables. In 60 (78.9%) patients who did not undergo upfront ASCT, high PD-1 expression was observed in 5 (8.3%) patients and they tended to associate with inferior survival (Figure 3a for OS and Figure 3b for PFS) although not statistically significant.

### Survival of patients with high PD-1 expression

We further analyzed survival of patients with high PD-1 expression to better characterize the patients with high PD-1 expression in PCNSL tumor microenvironment. The median survival of patients with high PD-1 expression was only 15.8 months (95% CI: 11.7–19.9) for OS and 10.4 months (95% CI: 3.3–17.5) for PFS. However, the median OS of patients with low PD-1 expression was not reached, and median PFS was 24.7 months (95% CI: 9.8–39.5) in patients with low PD-1. All the PCNSL patients with high PD-1 expression had DLBCL histology and two out of six patients showed positive serum EBV DNA among high PD-1 patients. Among six patients with high PD-1 expression, three patients achieved CR1 and five patients achieved CR after completion of primary chemotherapy.

Among these high PD-1 expressing patients, four patients died of disease progression, although four of whom received WBRT consolidation (median 4.0 [range 30.6–54.0] Gy) and one patient received upfront ASCT consolidation. Among four patients with high PD-1 who died of progression, two patients were intermediate-risk, one patient was low-risk, and one patient had missing data according to the IELSG scoring. Regarding MSKCC scoring, two patients belonged to low-risk, and the remaining two patients belonged to intermediate and high-risk, respectively. Additional characteristics of patients with high PD-1 expression are provided in Table 5.

**Table 5:** Clinical characteristics of six patients with high PD-1 expression

	Age		Gender		ECOG PS		LDH		Lesion		CSF protein		IELSG scoring		MSKCC scoring		PD-1, cells/ HPF		PD-L1, cells/ HPF		PD-L2, cells/ HPF		Initial treatment		Interim response		Final response after primary chemotherapy		Treatment after primary chemotherapy		OS (mon)		PFS (mon)		COD	
1	68		F		2		Elevated		Non-deep		Normal		3		2		92		10		0		MVP		CR1		CR		Consolidation WBRT		15.8		10.4		PD
2	34		F		1		Normal		Deep		Elevated		2		0		132		10		110		MVP		CR1		CR		Upfront ASCT		8.1		8.1		PD
3	59		F		1		Normal		Non-deep		Normal		0		1		230		7		5		MVD		Non- CR1		CR		Consolidation WBRT		34.5		15.2		Censored
4	64		M		1		Normal		Deep		N/A		N/A		1		150		18		200		MVD		Non- CR1		CR		Consolidation WBRT		17.9		17.9		PD
5	39		M		1		Normal		Deep		Normal		1		0		240		78		200		MVD		Non- CR1		PR		Salvage Chemotherapy		14.5		9.3		PD
6	60		M		1		Normal		Deep		Elevated		2		1		157		5		70		MVD		CR1		CR		Consolidation WBRT		21.8		16.2		Censored

Abbreviations: ECOG, Eastern Cooperative Oncology Group; PS, performance status; LDH, lactate dehydrogenase; CSF, cerebrospinal fluid; EBV, Epstein-Barr virus; IELSG, International Extranodal Lymphoma Study Group; MSKCC, Memorial Sloan Kettering Cancer Center; HPF, high power field; OS, overall survival; PFS, progression-free survival; COD, cause of death; F, Female; M, Male; DLBCL, diffuse large B-cell lymphoma; N/A, not available; MVP, methotrexate, vincristine, procarbazine and dexamethasone; MVD, methotrexate, vincristine and dexamethasone; CR1, complete response after initial two cycles of chemotherapy; CR, complete response; PR, partial response; ASCT, autologous stem-cell transplantation; WBRT, whole-brain radiotherapy; PD, progressive disease.

### Correlation of PD-1 expression with PD-L1 and PD-L2 expression levels

The association of PD-1 expression with PD-L1 and PD-L2 expression in tumor microenvironment of PCNSL is shown in Table 6. The level of PD-1 expression was significantly associated with the level of PD-L1 (*P* = 0.020) and PD-L2 (*P* < 0.001), respectively (Table 6).

**Table 6:** Correlation of PD-1 expression with PD-L1 and PD-L2

Parameters	Correlation coefficient	*P*
PD-L1	0.267	0.020
PD-L2	0.397	<0.001

## DISCUSSION

In this study, we found that high immunohistochemical PD-1 expression in biopsy specimen of patients with PCNSL at diagnosis was significantly associated with inferior OS and PFS.

Much information has been accumulated regarding the pathogenesis and tumor microenvironment of PCNSL [13]. However, in addition to the rarity of the disease, obtaining adequate numbers of specimens is difficult and thus prognostic roles of studied biomarkers have been unclear. Therefore, IELSG or MSKCC scoring is thus far considered the best available clinical tool for risk-stratifying PCNSL patients [2, 3], although biological prognostic markers that also consider the tumor microenvironment and that can be used as a therapeutic target are needed to improve prognostication and survival of patients with PCNSL. Although our group recently reported prognostic importance of CD68 expression in PCNSL microenvironment [6], the major limitation was regarding therapeutic implication as it is difficult to modulate immune response via CD68 positive macrophages in PCNSL tumor microenvironment.

In this regard, PD-1 is an attractive emerging therapeutic target because it has been shown to be expressed in various cancers as well as in hematologic malignancies [14, 15]. Regarding PD-1 expression in PCNSL, Berghoff *et al*. [16] were among the first to demonstrate PD-1 and PD-L1 expression in PCNSL. Of 20 PCNSL patient specimens, 2 (10.0%) showed moderate intensity and 1 (5.0%) had high intensity. Besides, Four *et al.* [17] reported expression of high PD-1 (2+) in 6.2% of patients with PCNSL. The proportions of high PD-1 expressing patients are similar to our study, as 7.9% of our patients expressed high PD-1. Compared to those studies, our study recruited more number of patients who were initially homogenously treated with HD-MTX-based chemotherapy. We also performed subgroup analysis according to the upfront ASCT, as the role of upfront ASCT in high-risk patients with PCNSL was emphasized [18].

One of our patient with high PD-1 expression died of progression even after receiving upfront ASCT. Interestingly, the poor prognosis of patients with high PD-1 expression was initially indistinguishable using the IELSG or MSKCC scoring systems; because among four high PD-1 expressing patients who died of disease progression, none of them belonged to the high-risk group according to IELSG scoring and only one of them belonged to the high-risk group regarding MSKCC scoring. However, if we knew that high PD-1 expression predicted poor survival in PCNSL, we would have been able to get more help regarding selection of treatment for these high-risk patients.

There were high expressions of PD-L1 and PD-L2 in 13.2% and 42.1% of our cohort, respectively. Regarding PD-L1 expression, our data also showed quite similar rate to that of Four *et al.* [17], as they reported 18.7% patients were PD-L1 high. However, these rates are relatively low compared to a recent study of PD-L1 expression in glioblastoma, which is a completely different disease but also the malignant case in the CNS [19].

PD-L1 expression might have been induced by genetic aberrations within tumor cells such as Hodgkin lymphoma and primary mediastinal large B-cell lymphoma, which harbor amplification of 9p24.1, a genomic region that encodes PD-L1 and PD-L2. Interestingly, gain of 9p24.1 was also observed in PCNSL [7]. Indeed, PCNSL is associated with immunodeficiency [1], and PCNSL might evade the immune system utilizing PD-1 pathway [9], resulting in poorer outcome compared to non-CNS disease. Our observation suggests a possible link between high PD-1 expression and poor survival, which could explain the more aggressive behavior associated with high PD-1 expression. Furthermore, we demonstrated significant correlation of PD-1 expression with PD-L1 and PD-L2, which is in line with previous studies [17, 20]. Therefore, our study might be used as a basis for future clinical trial targeting PD-1 pathway in PCNSL.

We acknowledge our limitation as the expression of PD-1 and PD-L1/PD-L2 have not been defined in specific cell type due to the lack of double staining with CD20, CD3 or other markers for macrophages. However, expression of PD-1 in tumor infiltrating lymphocytes and PD-L1 in tumor cells were distinguished with microscopic visualization by two independent experienced hematopathologists. Therefore, our claim that high PD-1 expression in tumor microenvironment of PCNSL is associated with poor survival is assuming that high PD-1 expression on tumor infiltrating lymphocytes in PCNSL tumor microenvironment is associated with poor survival of patients with PCNSL at diagnosis.

We also estimated PD-1, PD-L1, and PD-L2 expression visually followed by calculation of the mean number of positive cells/HPF and cut-off values have been chosen by AUC of receiver operating characteristic (ROC) curve for analytic purposes. However, we acknowledge the fundamental limitation to standardize the way of determining the optimal cut-off values for PD-1 and its ligands expression from tumor biopsy samples is difficult. Therefore, the predictive relevance of our method and determining optimal cut-off values remain to be validated in future studies.

In conclusion, we showed that high PD-1 expression in PCNSL tumor microenvironment is significantly associated with inferior survival. Better knowledge of the PD-1 pathway in PCNSL, along with future trials that include PD-1 based biological risk-stratification and therapeutic targeting, are necessary for this challenging disease.

## MATERIALS AND METHODS

### Patients

Seventy-six biopsy-proven PCNSL patients between December 2004 and March 2016 at Severance Hospital, South Korea for whom archived formalin-fixed and paraffin-embedded (FFPE) tissue blocks at initial diagnosis were available were retrospectively analyzed. The patient cohort was previously described [6]. All the patients included in this study were immunocompetent and did not have a history of immunosuppressive drug use nor malignancies other than PCNSL. We excluded human immunodeficiency virus-related PCNSL. The diagnosis was made histologically by surgical resection or stereotactic brain biopsy. All the patients received HD-MTX-based chemotherapy as an initial treatment homogenously without receiving steroid treatment before pathologic confirm.

PCNSL was stipulated as histologically confirmed NHL confined to the CNS [1]. Baseline clinical data were retrospectively collected including age, gender, IELSG prognostic score, MSKCC prognostic score, serum EBV positivity by quantitative polymerase chain reaction, serum human immunodeficiency virus positivity by enzyme immunoassay, type of treatment including consolidative upfront ASCT and WBRT, initial response to treatment, and survival.

The ECOG performance status was determined at the time of diagnosis. Pre-treatment evaluation included contrast enhanced magnetic resonance imaging of the brain, positron emission tomography-computed tomography to exclude systemic NHL, bone marrow aspiration and biopsy with histologic, cytologic, and immunocytologic examination, ocular examination including a slit lamp examination to distinguish ocular involvement, and lumbar puncture for cerebrospinal fluid (CSF) analysis, unless contraindicated. Elevated CSF protein concentration was defined as a level more than 45 mg/dL in patients younger than 60 years of age, and a level more than 60 mg/dL in patients older than 60 years of age [2]. Involvement of deep brain structures was defined as involvement of the periventricular regions, basal ganglia, brainstem, and cerebellum.

The IELSG scoring system [2], which consists of five prognostic factors that are associated with poor survival in PCNSL including age > 60 years, ECOG performance status > 1, elevated serum lactate dehydrogenase level, elevated CSF protein concentration, and involvement of deep brain structures, was assessed in 66 (86.8%) patients, as data regarding the CSF protein level were not available in 10 (13.2%) patients. The MSKCC scoring system [3], consisting of age ≥ 50 and Karnofsky performance score (KPS) ≥ 70, which are predictors of poor outcome in PCNSL, was evaluated in all patients.

All patients included in this study had received HD-MTX-based chemotherapy as an initial treatment, such as HD-MTX, vincristine, and dexamethasone (MVD, *n* = 41) or HD-MTX, procarbazine, and dexamethasone (MVP, *n* = 35). The MVD regimen consisted of HD-MTX 3.5 g/m^2^ D1 delivered intravenously (i.v.) with leucovorin rescue (15 mg every 6 hours, minimum 12 doses), started 24 hours after the MTX infusion, vincristine 1.4 mg/m^2^ (maximum 2 mg i.v.) D1, and dexamethasone 20 mg i.v. D1-5. The MVP regimen consisted of HD-MTX 3.0 g/m^2^ D1 i.v. with leucovorin rescue (15 mg every 6 hours, minimum 12 doses), started 24 hours after the MTX infusion, vincristine 1.4 mg/m^2^ (maximum 2 mg i.v.) D1, procarbazine 100 mg/m^2^ D1-14 given orally, and dexamethasone 20 mg i.v. D1-14. Tumor response was assessed according to the criteria of the International PCNSL Collaborative Group [21]. Treatment response was recorded as complete response1 (CR1), if the patients achieved a CR (disappearance of all enhancing abnormalities on brain magnetic resonance imaging) after initial induction HD-MTX-based chemotherapy. The institutional review board of Severance Hospital approved this study.

### Immunohistochemistry of PD-1, PD-L1, and PD-L2

Immunohistochemical staining for PD-1 (clone NAT105; dilution 1:100; Abcam, Cambridge, UK), PD-L1 (clone ab58810; dilution 1:100; Abcam, Cambridge, UK), and PD-L2 (clone MIH18; dilution 1:50; Sigma-Aldrich, St. Louis, MO, USA) using archived FFPE tissue slides were performed according to the protocols for automated immunohistochemistry using the Ventana Discovery XT automatic platform (Ventana Automated Systems, Tucson, AZ, USA).

### Quantitative assessment of PD-1, PD-L1, and PD-L2

The stained sections were screened at 40× magnification via Olympus BX51 microscope (Olympus, Deutschland GmbH, Hamburg, Germany; 40× objective, 10× ocular, and 0.55 mm ocular diameter) to identify the areas with the most abundant positive cells within the tumor area. Six representative fields with the highest staining tumor cells were selected at a magnification of 400× HPF. We analyzed 1.44 mm^2^ per case because the area of a single image was 0.24 mm^2^. PD-1 positive tumor-infiltrating mononuclear cells (i.e. lymphocytes and macrophages) and PD-L1/PD-L2 positive tumor cells were counted manually, and the mean numbers of positive cells/HPF were calculated. Blinded quantitative assessments were performed by two independent pathologists (SHK and W-IY). The cut-off values for high PD-1, PD-L1, and PD-L2 were evaluated by the AUC of the ROC curve, and we established cut-offs of 70 cells/HPF for PD-1 and 100 cells/HPF for PD-L1/PD-L2. Therefore, high PD-1 expression was defined as ≥ 70 cells/HPF, and high PD-L1/PD-L2 were defined as ≥ 100 cells/HPF.

### Statistical analysis

OS was defined as the time from initial diagnosis to death or last follow-up; PFS was defined as the time from initial diagnosis to relapse, disease progression, or death. Patient data were collected retrospectively until March 2016, and patients who were alive at the last follow-up contact were censored. OS and PFS were plotted using the Kaplan-Meier method and compared using the log-rank test. Analysis of categorical variables was performed using the chi-squared test or Fisher's exact test. The Wilcoxon rank-sum test was used for continuous variables. The Cox regression model was used in both univariate and multivariate analysis. All *P* values were two-sided, and *P* < 0.05 was considered statistically significant. Statistical analyses were performed using SPSS for Windows (Version 20.0, IBM Corp., Chicago, IL, USA).
